# Recent advances in the management of lymphangioleiomyomatosis

**DOI:** 10.12688/f1000research.14564.1

**Published:** 2018-06-18

**Authors:** Kai-Feng Xu, Xinlun Tian, Jay H Ryu

**Affiliations:** 1 Department of Respiratory Medicine, Peking Union Medical College Hospital, Peking Union Medical College and Chinese Academy of Medical Sciences, Beijing , China; 2 Division of Pulmonary and Critical Care Medicine, Mayo Clinic , Rochester, MN, USA

**Keywords:** Angiomyolipoma, Chylothorax, Lung transplantation, Lymphangioleiomyomatosis, Pneumothorax, Pulmonary rehabilitation, Rapamycin, Sirolimus, Tuberous sclerosis complex

## Abstract

Lymphangioleiomyomatosis is a rare disorder that predominantly affects women and is characterized by progressive cystic changes in the lung, leading to gradually worsening shortness of breath and lung function impairment. Pleural complications such as pneumothorax and chylothorax commonly occur in these patients. Lymphangioleiomyomatosis can occur as a form of lung involvement in tuberous sclerosis complex or as a sporadic form (without tuberous sclerosis complex). Etiology in both forms of this disease centers on mutations in the tuberous sclerosis genes. Advances in our understanding of the regulatory role of tuberous sclerosis gene products (hamartin/tuberin) in the mechanistic target of rapamycin (mTOR) signaling pathway have led to the identification of effective therapy (mTOR inhibitors) for a rare disorder, once considered uniformly fatal. Here, we summarize the evolution of current concepts regarding lymphangioleiomyomatosis with an emphasis on recent advances and unresolved issues.

## Introduction

Lymphangioleiomyomatosis (LAM) is a rare disease that occurs primarily in women and manifests as diffuse cystic changes in the lung ^1, 2^. The presenting symptoms may include dyspnea or chest pain (often related to spontaneous pneumothorax) or both ^3, 4^. Some patients may present for evaluation of imaging abnormalities, including cystic lung disease, pleural effusion, or renal tumor (associated renal angiomyolipomas, or AML) in the absence of symptoms.

LAM can be sporadic or occur in patients with a heritable disorder, tuberous sclerosis complex (TSC), and had been considered a lethal disorder with no effective therapy. In 2010, the European Respiratory Society (ERS) published the first clinical guidelines for the diagnosis and management of LAM ^3^. In 2011, sirolimus (rapamycin) therapy was demonstrated to stabilize lung function in patients with LAM ^5^. Currently, LAM is considered a low-grade, metastasizing neoplasm ^1^. Advances in our understanding of the disease process in recent years have profoundly changed the management of LAM and the prognosis for affected patients. Table 1 outlines the major events that have influenced clinical practice in recent years ^3, 5– 11^. In this review, we aim to summarize the recent evolution of concepts concerning the state-of-the-art management of LAM.

**Table 1.** Recent landmark events in the management of lymphangioleiomyomatosis.

Year	Events
2008	Sirolimus is demonstrated to induce regression of TSC- or sporadic LAM-associated renal angiomyolipomas and improve spirometric measurements in some patients with LAM ^6^.
2010	The European Respiratory Society publishes the first clinical guidelines for the diagnosis and management of LAM ^3^.
2011	Clinical trial demonstrates the efficacy and safety of sirolimus therapy in patients with sporadic and TSC-associated LAM ^5^.
2013	Updated international consensus statement on the diagnosis and management of TSC is published ^7, 8^.
2014	Sirolimus is approved for LAM treatment in Japan.
2015	Sirolimus is approved for LAM treatment in the USA.
2015	LAM is listed in the 2015 World Health Organization Classification of Lung Tumors ^9^.
2016	ATS/JRS LAM clinical practice guidelines are published ^10^.
2017	ATS/JRS LAM expanded clinical practice guidelines are published ^11^.

ATS, American Thoracic Society; JRS, Japanese Respiratory Society; LAM, lymphangioleiomyomatosis; TSC, tuberous sclerosis complex.

## Sirolimus therapy for lymphangioleiomyomatosis

Sirolimus, or rapamycin, inhibits the mechanistic target of rapamycin (mTOR) signaling pathway and currently plays a central role in the treatment of LAM. The molecular pathogenesis of LAM is based on *TSC2* gene mutations and their effect on the mTOR pathway, which is normally controlled by the protein complex (hamartin/tuberin) encoded by *TSC1* and *TSC2* genes, respectively ^12, 13^. When the *TSC2* or *TSC1* gene is mutated, the resulting protein complex fails in its role as an upstream negative regulator of mTOR and results in its constitutive activation ^14^. This mTOR activation, in turn, results in unregulated cell growth.

Based on the discovery of *TSC2* mutation in patients with sporadic LAM and the mechanism of action of sirolimus, clinical trials were initiated assessing the efficacy of sirolimus therapy for not only TSC-related tumors but also LAM. In 2008, Bissler *et al*. published the first clinical trial on the use of sirolimus for renal AMLs in patients with TSC as well as sporadic LAM ^6^. In the 12-month treatment period, sirolimus significantly reduced the size of AMLs. The pulmonary function measurements for the 11 LAM patients in the study improved as well. Subsequently, a randomized placebo-controlled study of sirolimus for LAM—Multicenter International Lymphangioleiomyomatosis Efficacy of Sirolimus (MILES) study—enrolled 89 patients ^5^. The forced expiratory volume at 1 second (FEV _1_) increased or remained stable during 12 months of treatment. The level of serum vascular endothelial growth factor-D (VEGF-D), a biomarker reflecting disease activity, decreased significantly during treatment with sirolimus.

Sirolimus has now been widely administered to patients with LAM. Multiple additional clinical studies on the use of sirolimus for LAM have been published, although the sample sizes of the studies were relatively small because of the rarity of this disease ^15^. Overall, sirolimus therapy was demonstrated to stabilize lung function, improve quality of life, reduce the size of chylothorax, decrease the volume of renal AMLs, and decrease serum VEGF-D level ^16– 23^.

### Indications for sirolimus therapy in lymphangioleiomyomatosis

Diffuse cystic lung disease can be seen in disorders other than LAM, and the differential diagnosis of diffuse cystic lung disease has been reviewed elsewhere ^2, 24^. The diagnosis of LAM is classified as “definite”, “probable”, or “possible” according to the criteria outlined in the 2010 ERS guidelines ^3^. The diagnostic criteria for definite LAM were updated recently in the American Thoracic Society/Japanese Respiratory Society (ATS/JRS) guidelines published in 2017. Accordingly, a definite diagnosis of LAM can be established when a patient has compatible clinical and radiological findings—diffuse cystic lung disease depicted on high-resolution computed tomography (CT) of the chest—combined with one of the following features: presence of TSC, renal AMLs, chylothorax, lymphangioleiomyomas, elevated serum VEGF-D level, presence of LAM cells demonstrated in effusions or lymph nodes, or histopathological confirmation by biopsy of lung or extrapulmonary lesion. Patients with compatible clinical and radiological features can be diagnosed with “probable” LAM in the absence of additional diagnostic criteria.

Sirolimus therapy is currently recommended for patients with a definite diagnosis of LAM who manifest an abnormal (FEV _1_ of less than 70% of the predicted value) or declining pulmonary function ^10^. Other indications for the use of sirolimus in patients with LAM may include symptomatic chylothorax or chylous ascites ^10^, renal AMLs ^6^, retroperitoneal or pelvic lymphangioleiomyomas, and other TSC-related lesions ^15^.

### Optimal dosage of sirolimus therapy in lymphangioleiomyomatosis

Although optimal dosage of sirolimus for LAM has not been defined, the currently favored dosage for LAM is 1–2 mg per day (“low dose”) to achieve a serum trough level of around 5 ng/mL, which is on the lower end of the original target serum trough level in the MILES trial (that is, between 5 and 15 ng/mL). In a retrospective analysis of 98 patients with LAM treated with this dosing regimen at Peking Union Medical College Hospital, the majority of patients achieved a trough serum level of 5–10 ng/mL while about 20% of patients attained a serum trough level of less than 5 ng/mL ^16^. A study involving 15 patients in Japan suggested that an even lower dosing regimen that achieves a serum trough level of less than 5 ng/mL may provide treatment effects equivalent to those of higher dosing regimens ^25^. Another study reported no association between the rate of change in FEV _1_ and serum sirolimus level ^23^.

We suggest monitoring patients with LAM for progression of the disease while on sirolimus therapy as well as assessing for adverse drug-related effects. Although minimal effective dosage is preferred, higher doses of sirolimus may be needed in patients who continue to manifest disease progression. Table 2 outlines the suggested monitoring schedule for LAM patients on sirolimus therapy ^26^.

**Table 2.** Suggested schedule of monitoring assessments for lymphangioleiomyomatosis patients on sirolimus therapy.

Monitoring assessments	Prior to therapy	Initial 3 months	Every 6–12 months	In case of clinical worsening
Clinical	✓	✓	✓	✓
Pulse oximetry	✓	✓	✓	✓
Six-minute walking distance (6MWD)	✓		✓	✓
St George Respiratory Questionnaire	✓		✓	✓
Pulmonary function test	✓		✓	✓
Serum vascular endothelial growth factor-D (VEGF-D) level	✓		✓ ^a^	✓ ^a^
High-resolution computed tomography (HRCT) of chest	✓		✓ ^b^	✓ ^a^
Computed tomography/magnetic resonance imaging of abdomen and pelvis	✓		✓ ^c^	✓ ^c^
Sirolimus concentration		✓ ^d^	✓	✓
Sirolimus safety ^e^		✓	✓	✓

a Optional depending on the clinical requirements.

b HRCT of the chest can be assessed every 12 months or as clinically indicated.

c If abnormal at baseline or prior to therapy and as clinically indicated.

d The sirolimus dosage should be adjusted during the initial 3 months of treatment, if needed, and when necessary (for example, change in patient’s other medications that may influence serum sirolimus level).

e Including symptoms (rash, oral ulcer, menses, and so on) and laboratory investigations (lipid panel and so on).

Modified from Xu and Lo ^26^.

### Safety of sirolimus therapy in lymphangioleiomyomatosis

Sirolimus is generally well tolerated in patients with LAM. Common side effects of sirolimus therapy in LAM include acne-like rash, oral ulcer, irregular menses, peripheral edema, diarrhea, hyperlipidemia, and liver enzyme elevations. Sirolimus-associated pneumonitis is rare but can be severe ^21^. Pulmonary infections were not increased in patients with LAM treated with sirolimus ^5^. The long-term safety profile of sirolimus therapy has not yet been fully defined.

### Sirolimus treatment failure

Not all patients with LAM respond to sirolimus therapy. Currently, there is no consensus regarding the definition of treatment failure in LAM. Additionally, alternative therapeutic intervention for such cases is lacking. A phase I trial has been conducted using the combination of sirolimus and hydroxychloroquine (an autophagy inhibitor) and demonstrated the safety of this combination therapy ^27^. A phase II study of aromatase inhibitor, letrozole, showed this mode of therapy to be safe and well tolerated but, owing in part to under-enrollment of study participants, failed to show efficacy ^28^. Future studies are needed to clarify the definition of sirolimus treatment failure, underlying mechanisms, and optimal management strategies for affected patients.

## Pulmonary rehabilitation

Pulmonary rehabilitation is a standard practice for patients with chronic lung diseases ^29^. It can reduce dyspnea, increase exercise capacity, and improve quality of life. A controlled clinical trial consisting of 40 patients with LAM examined the effects of pulmonary rehabilitation ^30^. The program included twice-weekly 1-hour sessions of aerobic exercise on a treadmill and muscle strength training along with education for a period of 3 months. The pulmonary rehabilitation group exhibited improved exercise endurance time, quality of life, 6-minute walking distance, and peak oxygen consumption. No cases of pneumothorax or other serious events occurred during exercise. These results suggest that a pulmonary rehabilitation program should be employed in dyspneic patients with LAM. A study of yoga therapy in patients with LAM is ongoing in China (ChiCTR-OON-17012748, http://www.chictr.org.cn/showproj.aspx?proj=21738).

## Lung transplantation

Lung transplantation is a treatment option for patients with advanced LAM ^3^. Similar experiences have been reported from multiple countries on the use of lung transplantation in LAM ^31– 36^. The ERS guidelines recommended that patients with LAM be considered for lung transplantation when they reach a New York Heart Association (NYHA) functional class of III or IV with severely impaired lung function and exercise capacity ^3^. Prior pleurodesis and thoracic surgical procedures are associated with higher rates of bleeding and re-exploration but are not contraindications to lung transplantation ^11, 37^. The actuarial survival of lung transplant recipients with LAM is 65% at 5 years ^3^.

Sirolimus is a potent immunosuppressant and is commonly used in solid organ transplant recipients to prevent organ rejection. There have been reports of impaired wound healing and wound dehiscence associated with sirolimus therapy ^38– 40^. Given these concerns, questions have arisen regarding whether sirolimus therapy should be discontinued when patients with LAM are on a waiting list for lung transplantation and the safety of including sirolimus in the post-transplant immunosuppressive regimen.

When patients are on a waiting list for lung transplantation, most lung transplant programs advise patients to discontinue sirolimus therapy ^39^. However, the cessation of sirolimus therapy introduces the risk of disease progression and worsening in lung function in patients with LAM. It has been suggested that keeping patients on sirolimus therapy, particularly at a low-dose regimen, is likely safe for patients awaiting lung transplantation ^39^. Nonetheless, a consensus exists that sirolimus not be used in the immediate post-lung transplant period to minimize the risk of bronchial anastomotic dehiscence. Long-term benefits of reinitiating sirolimus therapy after bronchial anastomotic healing has occurred remain to be determined. These potential benefits may include prevention of LAM recurrence in the transplanted lung, reducing the risk of pleural complications (for example, chylothorax), and control of extrapulmonary manifestations (for example, renal AMLs).

## Other management issues in lymphangioleiomyomatosis

### Pneumothorax

Pneumothorax occurs commonly in patients with LAM. A National Heart, Lung, and Blood Institute (NHLBI) registry study of 230 patients with LAM reported pneumothorax to have occurred in 55.5% of subjects at enrollment into the study ^4^. Recurrence of pneumothorax is also common, occurring on average 4.4 times among those with a history of pneumothorax ^4^. Because the recurrence rate is high, current guidelines recommend pleurodesis at the time of the first episode of pneumothorax ^3, 11^. However, pleurodesis in patients with LAM has limited efficacy and the recurrence rate of pneumothorax is reported to range between 18% and 32% ^11, 41^. Therefore, more reliable methods to reduce the risk of recurrent pneumothorax in these patients have been sought. Kurihara *et al*. reported a new surgical technique using oxidized regenerated cellulose mesh to wrap the visceral pleura in a procedure called “total pleural covering” (TPC) ^42^. They retrospectively analyzed 43 LAM patients who underwent the TPC procedure (54 hemithoraces), 11 of whom required bilateral lung surgeries. A Kaplan-Meier estimate of recurrence-free hemithorax was 80.8% at 2.5 years.

### Chylothorax

Chylothorax occurs in about 7–10% of patients with LAM ^4, 43, 44^. The clinical effects and prognosis associated with chylothorax vary considerably ^43, 45^. Several different types of lymphatic abnormalities associated with chylothorax have been identified by CT and lymphangiography ^46^. Iliac or retroperitoneal lymphatic vessel dilation and obstruction of the thoracic duct are most commonly observed. Although the majority of physicians believe that LAM patients with chylothorax should be provided a low-fat or fat-free diet, the use of such dietary maneuvers requires monitoring of the nutritional status to avoid malnutrition ^3^. Observation or drainage by thoracentesis may be sufficient for patients with a small chylothorax ^3^. Prior to sirolimus therapy, various surgical techniques, including pleurodesis, serial thoracenteses, and thoracic duct ligation, were employed in patients with varying degrees of efficacy ^43, 47, 48^.

Currently, the most effective management for chylothorax associated with LAM is considered sirolimus therapy. Initial reports on the beneficial effects of sirolimus therapy in managing chylothorax associated with LAM were published in 2008 ^49, 50^. Subsequent studies have confirmed the effectiveness of sirolimus therapy on controlling chylothorax ^16, 25, 51, 52^.

### Renal angiomyolipoma

Renal AMLs are found in about 30% of patients with sporadic LAM and 90% of those with TSC-related LAM ^4^. Previously, the management of renal AMLs included embolization therapy or nephron-sparing surgery for tumors larger than 4 cm ^3^. Clinical practice has changed since the advent of mTOR inhibitor therapy (that is, sirolimus or everolimus) for LAM and TSC-related manifestations. It is now well established that sirolimus effectively reduces the size of renal AMLs. In a report from Bissler *et al*., sirolimus reduced the volume of renal AMLs by about half after 1 year of treatment ^6^. In a randomized placebo-controlled trial of everolimus for renal AMLs associated with sporadic LAM or TSC (EXIST-2), 42% of patients achieved a response defined as 50% reduction of tumor volume ^53^. A total of 58% of 112 patients achieved a response during a 4-year extension period of the study, while none of the treated patients experienced bleeding of the tumor or needed nephrectomy ^54^. Other studies have confirmed the effectiveness of sirolimus therapy for renal AMLs ^16, 17, 22^.

## Pulmonary hypertension

Pulmonary hypertension (PH) can be seen in patients with LAM with an estimated prevalence of 7% to 8% ^55– 57^. PH is usually mild to moderate in severity but can be associated with significant impact on functional capacity ^55– 57^. The mechanisms underlying PH may include hypoxemia (including exertion-related oxygen desaturation) and pulmonary vascular remodeling. Hemodynamic parameters tend to correlate with pulmonary function, especially FEV _1_, diffusion capacity of the lung for carbon monoxide (DLCO), and alveolar-arterial oxygen (PA-aO _2_) gradient. A recent report suggests that PH may improve with sirolimus therapy ^58^.

### Air travel

Ambient barometric pressure change that occurs during air travel has raised concerns regarding the risk of pneumothorax during flight for patients with cystic lung disease such as LAM. A survey study of 327 patients with LAM reported a rate of 2.2% for pneumothorax in flight ^59^. Other reported symptoms during air travel included chest pain, dyspnea, hypoxemia, nausea, dizziness, fatigue, headache, hemoptysis, and anxiety. Another study, of 281 patients with LAM, found a pneumothorax rate of 1.1% per flight ^60^.

Although the incidence of pneumothorax associated with air travel is low, advice regarding air travel should be formulated carefully on an individual basis ^61^. Patients with existing pneumothorax should avoid air travel. Patients who have recovered from pneumothorax or thoracic surgery should delay air travel for a period of a few weeks, until full resolution and healing have been achieved. Patients with symptoms of chest pain, severe dyspnea, or low oxygen saturation should be medically evaluated in advance of planned air travel.

### Pregnancy

LAM-associated complications, including pneumothorax and chylothorax, may occur during pregnancy, and concerns have been raised regarding possible association of pregnancy with progression of LAM ^62, 63^. Women with LAM have also been reported to experience worse pregnancy outcomes, including a greater number of premature births and miscarriages ^62, 63^.

Women with LAM should be informed of these increased risks associated with pregnancy ^3^. Patients with mild or moderate LAM will likely tolerate pregnancy better compared with those with more severe disease. The decision to proceed to pregnancy is a personal one that is made on an individual basis after assessing the severity of LAM, potential risks, and other options to pregnancy. During pregnancy, the patient should be monitored carefully by a multidisciplinary team that can optimally manage a high-risk pregnancy.

Sirolimus is included in the C risk category (risk to fetus has not been ruled out, and adverse effect to the fetus has been shown in animal studies but no adequate studies of humans) for US Food and Drug Administration pregnancy labeling. There are reports of successful pregnancy without teratogenicity in solid organ transplant recipients receiving sirolimus. Currently, initiation of contraception is recommended before starting sirolimus therapy and continued for 12 weeks after discontinuation.

### Avoidance of exogenous estrogen

Several lines of observation suggest that estrogen promotes the growth and spread of LAM cells. For example, LAM occurs mostly in women and seems to manifest slowing of disease progression after menopause. There are *in vitro* data demonstrating that neoplastic potential and survival of LAM cells are enhanced by estrogen ^64^. Thus, it is generally advised that exogenous estrogen exposure (for example, estrogen replacement therapy) be avoided for patients with LAM.

## Summary

Not long ago, LAM was considered a uniformly fatal lung disease for those who became afflicted with this rare and poorly understood condition. Remarkable progress has occurred, particularly over the past decade, leading to effective medical therapy that prevents progression of disease for most patients. There are unanswered questions regarding the long-term efficacy and safety of mTOR inhibitor therapy for the treatment of LAM. In addition, there is a need to identify other medical treatment options for those patients who experience disease progression despite mTOR inhibition.
